# Supplementary material for: Analysis of the Impact of Disease Acceptance, Demographic, and Clinical Variables on Adherence to Treatment Recommendations in Elderly Type 2 Diabetes Mellitus Patients
Source: Int J Environ Res Public Health. 2021 Aug 16;18(16):8658. doi: 10.3390/ijerph18168658 (PMC8391118; doi:10.3390/ijerph18168658)
Supplement: Supplementary file 1 [file ijerph-18-08658-s001.zip › 1. SCODI.pdf]

INWENTARZ SAMOOPIEKI W CUKRZYCY (ang. SELF-CARE OF DIABETES INVENTORY - SCODI)

Proszę pomyśleć o wykonywanych czynnościach dotyczących samoopieki oraz towarzyszących im odczuciach w ciągu ostatniego miesiąca.

SEKCJA A

Poniżej zostały wymienione niektóre zachowania, które osoba z cukrzycą może wprowadzić w celu utrzymania zdrowia i dobrego samopoczucia. Proszę wskazać jak często wprowadzasz te zachowania.

(zakreśl jedną liczbę)

		**Nigdy**				**Zawsze**
**1.**	Utrzymywanie aktywnego stylu życia (np. spacery, wychodzenie z domu, prowadzenie aktywności)?	1	2	3	4	5
**2.**	Wykonywanie ćwiczeń fizycznych przez 2,5 godziny każdego tygodnia (np. pływanie, chodzenie na siłownię, jazda na rowerze, spacery)?	1	2	3	4	5
**3.**	Stosowanie zbilansowanej diety i spożywanie węglowodanów (makaron, ryż, cukry, chleb), białka (mięso, ryby, rośliny strączkowe), owoców i warzyw?	1	2	3	4	5
**4.**	Unikanie spożycia soli i tłuszczów (np. ser, wędliny, słodycze, czerwone mięso?	1	2	3	4	5
**5.**	Ograniczenie spożycia alkoholu (nie więcej niż 1 kieliszek wina dziennie dla kobiet i 2 kieliszki dziennie dla mężczyzn)?	1	2	3	4	5
**6.**	Unikanie zachorowania (np. mycie rąk, korzystanie z zalecanych szczepień ochronnych)?	1	2	3	4	5
**7.**	Unikanie palenia papierosów i dymu tytoniowego?	1	2	3	4	5
**8.**	Dbanie o własne stopy (np. mycie i suszenie skóry, nawilżanie, używanie odpowiednich skarpet)?	1	2	3	4	5
**9.**	Utrzymywanie prawidłowej higieny jamy ustnej (mycie zębów co najmniej 2 razy dziennie, używanie płynów do płukania i nici dentystycznych)?	1	2	3	4	5
**10.**	Uczęszczanie na wizyty do swojego lekarza?	1	2	3	4	5
**11.**	Systematyczne kontrolowanie stanu zdrowia (np. badania krwi i moczu, USG, badanie wzroku)?	1	2	3	4	5
**12.**	Wiele osób ma problemy z przyjmowaniem wszystkich przepisanych leków. Czy zażywasz wszystkie swoje leki zgodnie z zaleceniami lekarza (proszę rozważyć również przyjmowanie insuliny, jeśli jest przepisana)?	1	2	3	4	5

SEKCJA B

Poniżej zostały wymienione niektóre zachowania, które osoba z cukrzycą może praktykować w celu monitorowania przebiegu cukrzycy. Proszę wskazać jak często praktykujesz te zachowania.

(zakreśl jedną liczbę)

		**Nigdy**				**Zawsze**
**13.**	Regularne kontrolowanie poziomu glikemii (cukru we krwi)?	1	2	3	4	5
**14.**	Systematyczne kontrolowanie masy ciała?	1	2	3	4	5
**15.**	Skrupulatne kontrolowanie ciśnienia krwi?	1	2	3	4	5
**16.**	Prowadzenie zapisów poziomu glikemii (cukru) w dzienniku lub notatniku?	1	2	3	4	5
**17.**	Codzienne monitorowanie stanu stóp i obserwacja występowania, zaczerwienienia , pęcherzy lub ran?	1	2	3	4	5
**18.**	Zwracanie uwagi na objawy wysokiego poziomu glikemii (cukru we krwi) (wzmożone pragnienie, częste oddawanie moczu) lub niskiego poziomu glikemii (osłabienie, wzmożona potliwość)?	1	2	3	4	5

Uwaga. Jeśli w ostatnim miesiącu prowadzono ciągły pomiar stężenia glukozy we krwi, odpowiedz „5" przy nr 13 i 16.

Podczas gdy ostatni raz wystąpiły objawy:

		**Nie rozpoznałem (łam) objawów**	**Rozpoznałem po jakimś czasie**			**Bardzo szybko**
**19.**	Jak szybko rozpoznałeś(łaś), że masz objawy?	0	1	2	3	4	5
**20.**	Jak szybko rozpoznałeś(łaś), że objawy dotyczą cukrzycy?	0	1	2	3	4	5

SEKCJA C

Poniżej zostały wymienione niektóre zachowania, które osoba z cukrzycą może podejmować, aby poprawić poziom cukru we krwi, gdy jest zbyt wysoki lub zbyt niski. Jak często podejmujesz te działania, gdy wystąpią objawy lub kiedy poziom cukru we krwi jest poza zakresem normy?

(zakreśl jedną liczbę)

		**Nigdy**				**Zawsze**
**21.**	Sprawdzasz poziom glikemii (cukru we krwi), gdy poczujesz objawy takie jak pragnienie, częste oddawanie moczu, osłabienie, pocenie się, niepokój?	1	2	3	4	5
**22.**	Kiedy masz nieprawidłowy poziom glikemii (cukru we krwi), robisz notatki o wydarzeniach, które mogły być tego przyczyną?	1	2	3	4	5
**23.**	Kiedy masz nieprawidłowy poziom glikemii (cukru we krwi), pytasz o poradę członka rodziny lub przyjaciela?	1	2	3	4	5
**24.**	Kiedy masz objawy i odkrywasz, że poziom glikemii (cukru we krwi) jest zbyt niski, to czy jesz lub pijesz produkty z cukrem, aby rozwiązać ten problem?	1	2	3	4	5
**25.**	Jeśli dowiadujesz się, że poziom glikemii (cukru we krwi) jest zbyt wysoki, to czy dostosowujesz dietę, aby poprawić tę kwestię?	1	2	3	4	5
**26.**	Jeśli okazuje się, że poziom glikemii (cukru we krwi) jest zbyt wysoki, to czy regulujesz podejmowaną aktywność fizyczną, aby to skorygować?	1	2	3	4	5
**27.**	Po przeprowadzeniu działań mających na celu uregulowanie nieprawidłowego poziomu glikemii (cukru we krwi), sprawdzasz ponownie poziom glikemii (cukru we krwi), aby ocenić, czy podjęte działania były skuteczne?	1	2	3	4	5
**28.**	Jeśli dowiadujesz się, że poziom glikemii (cukru we krwi) jest bardzo niski lub bardzo wysoki, to czy dzwonisz po poradę do swojego lekarza?	1	2	3	4	5

Czy przyjmujesz insulinę?

- ☐ Tak
- ☐ Nie

Jeśli tak, proszę o udzielenie odpowiedzi na poniższe pytanie:

(zakreśl jedną liczbę)

		**Nigdy**				**Zawsze**
**29.**	Jeśli okaże się, że stężenie glikemii (cukru we krwi) jest zbyt wysokie lub zbyt niskie, to czy dostosowujesz dawkę insuliny w sposób sugerowany przez lekarza?	1	2	3	4	5

SEKCJA D

Osoby z cukrzycą muszą rozwijać swoje umiejętności w zakresie szczególnego dbania o siebie i utrzymania dobrego stanu zdrowia. Na ile pewnie czujesz się wykonując następujące czynności?

(zakreśl jedną liczbę)

		**Całkowicie niepewnie**				**Bardzo pewnie**
**30.**	Zapobiegasz zbyt wysokim lub zbyt niskim poziomom glikemii (cukru we krwi) i objawom z tym związanym.	1	2	3	4	5
**31.**	Postępujesz zgodnie z zaleceniami w kwestii odżywiania i aktywności fizycznej.	1	2	3	4	5
**32.**	Zażywasz leki w odpowiedni sposób (w tym insulinę, jeśli została przepisana).	1	2	3	4	5
**33.**	Postępujesz zgodnie z założonym planem leczenia, nawet jeśli jest trudny do zrealizowania.	1	2	3	4	5
**34.**	Monitorujesz poziom glikemii (cukru we krwi) tak często, jak polecił to Twój lekarz.	1	2	3	4	5
**35.**	Rozumiesz, czy Twój poziom glikemii (cukru we krwi) jest dobry, czy nie.	1	2	3	4	5
**36.**	Rozpoznajesz objawy zbyt niskiego poziomu glikemii (cukru we krwi).	1	2	3	4	5
**37.**	Monitorujesz przebieg cukrzycy, nawet gdy jest to trudne do wykonania.	1	2	3	4	5
**38.**	Podejmujesz działania, aby uregulować poziom glikemii (cukru we krwi) i złagodzić objawy.	1	2	3	4	5
**39.**	Oceniasz, czy podjęte przez Ciebie działania skutecznie poprawiają poziom glikemii (cukru we krwi) i łagodzą objawy.	1	2	3	4	5
**40.**	Utrzymujesz działania mające na celu poprawę poziomu glikemii (cukru we krwi), nawet gdy jest to trudne.	1	2	3	4	5

Dziękujemy za wypełnienie ankiety!

Wersja oryginalna (Ausili D, Barbaranelli C, Rossi E, Rebora P, Fabrizi D, Coghi C, Luciani M, Vellone E, Di Mauro S, Riegel B.)
Polska adaptacja: Uchmanowicz I., Krzemińska S.
