# Supplementary material for: Analysis of the Impact of Disease Acceptance, Demographic, and Clinical Variables on Adherence to Treatment Recommendations in Elderly Type 2 Diabetes Mellitus Patients
Source: Int J Environ Res Public Health. 2021 Aug 16;18(16):8658. doi: 10.3390/ijerph18168658 (PMC8391118; doi:10.3390/ijerph18168658)
Supplement: Supplementary file 1 [file ijerph-18-08658-s001.zip › 2.ACDS_PL-2.pdf]

<u>Skala adherence w chorobach przewlekłych</u>
The Adherence in Chronic Diseases Scale (ACDS)

Autor: Aldona Kubica

Poniżej zamieszczono zestaw 7 pytań wraz z odpowiedziami. Proszę oceń, która z odpowiedzi najlepiej odzwierciedla Twoje zachowania, Twoją sytuację oraz Twoje poglądy. Proszę o udzielenie szczerych odpowiedzi poprzez zaznaczenie właściwej znakiem X.

---

1. Czy zawsze pamiętasz o przyjmowaniu wszystkich leków zgodnie z zaleceniami lekarza?
   A. Zawsze
   B. Prawie zawsze
   C. Czasami
   D. Prawie nigdy
   E. Nigdy

2. Czy okresowo zmieniasz dawkowanie leków bez porozumienia ze swoim lekarzem?
   A. Nigdy
   B. Bardzo rzadko
   C. Czasami
   D. Często
   E. Nie stosuję się wcale do zalecanego dawkowania

3. Czy dostosowujesz przyjmowanie leków do swojego samopoczucia?
   A. Stosuję wszystkie leki regularnie, niezależnie od samopoczucia,
   B. Obniżam dawkę niektórych leków, gdy czuję się bardzo dobrze
   C. Opuszczam dawki niektórych leków, gdy czuję się bardzo dobrze
   D. Okresowo odstawiam niektóre leki, gdy czuję się bardzo dobrze
   E. Zaprzestaję przyjmowania wszystkich leków, gdy czuję się bardzo dobrze

4. Gdy pojawiają się objawy uboczne związane z przyjmowaniem leków (np. bóle żołądka, bóle wątroby, wysypka, brak apetytu, obrzęki) wówczas
   A. Natychmiast zgłaszam się do lekarza
   B. Zmniejszam dawkę leku i staram się przyspieszyć planowaną wizytę u lekarza
   C. Odstawiam lek i staram się przyspieszyć planowaną wizytę u lekarza
   D. Odstawiam lek i czekam na planową wizytę u lekarza
   E. Odstawiam wszystkie leki i czekam na planową wizytę u lekarza

5. Czy uważasz, że wszystkie leki, które przyjmujesz są potrzebne dla zachowania dobrego stanu zdrowia?
   A. Tak, uważam, że wszystkie leki pomagają mi utrzymać dobry stan zdrowia
   B. Większość leków, które przyjmuję pomagają mi utrzymać dobry stan zdrowia
   C. Tylko niektóre leki, które przyjmuję pomagają mi utrzymać dobry stan zdrowia
   D. Niektóre zapisane leki mi pomagają, ale inne szkodzą
   E. Większość leków stosowanych przez dłuższy czas szkodzi mojemu zdrowiu

6. Czy Twój lekarz pyta Cię o niedogodności związane z przyjmowaniem leków?
   A. Tak, za każdym razem
   B. Tak, zazwyczaj
   C. Czasami
   D. Rzadko
   E. Nigdy

7. Czy szczerze odpowiadasz na pytania lekarza dotyczące przyjmowania leków?
   A. Tak, zawsze
   B. Prawie zawsze
   C. Staram się być szczery, ale czasami trudno przyznać się, że nie przestrzegam zaleceń
   D. Czasami tak, czasami nie
   E. Nie, to moja prywatna sprawa

Punktacja
A – 4
B – 3
C – 2
D – 1
E – 0

SUMA UZYSKANYCH PUNKTÓW: …………………

**Liczba punktów**	**Normy centylowe**
≤20	poziom niski
21-26	poziom średni
≥27	poziom wysoki

<u>Skala adherence w chorobach przewlekłych</u>
The Adherence in Chronic Diseases Scale (ACDS)

Autor: Aldona Kubica

Minimalna liczba punktów: 0
Maksymalna liczba punktów: 28

Interpretacja wyników:

Wysoki adherence
Średni adherence
Niski adherence

Założenie ACDS: jedynie wysoki adherence wg. ACDS odzwierciedla dobrą realizację planu terapeutycznego w zakresie farmakoterapii.

Opis i zastosowanie

Skala adherence w chorobach przewlekłych zawiera 7 pytań wraz z proponowanymi zestawami 5 odpowiedzi do każdego pytania. Pytania odnoszą się do zachowań bezpośrednio determinujących adherence (pytania 1-5), oraz do sytuacji i poglądów, które mogą pośrednio wpływać na adherence (pytania 6-7).
Skala jest przeznaczona do badania osób dorosłych leczonych z powodu chorób przewlekłych. Narzędzie to ma nie tylko odzwierciedlać rzeczywistą realizację planu terapeutycznego w zakresie farmakoterapii, ale także wskazywać na mechanizmy determinujące adherence pacjentów.
Wyniki mogą być pomocne w podejmowaniu działań na rzecz poprawy regularności przyjmowania leków w praktyce klinicznej.
