# Supplementary material for: Analysis of the Impact of Disease Acceptance, Demographic, and Clinical Variables on Adherence to Treatment Recommendations in Elderly Type 2 Diabetes Mellitus Patients
Source: Int J Environ Res Public Health. 2021 Aug 16;18(16):8658. doi: 10.3390/ijerph18168658 (PMC8391118; doi:10.3390/ijerph18168658)
Supplement: Supplementary file 1 [file ijerph-18-08658-s001.zip › 3.SKALA AKCEPTACJI CHOROBY.pdf]

SKALA AKCEPTACJI CHOROBY (AIS)

......................płeć M K wiek ……………data badania…………..

Proszę ustosunkować się do każdego stwierdzenia zaznaczając na skali od 1 do 5 liczbę, która najlepiej określa Pana/i obecny stan. Swój wybór należy zaznaczyć otaczając kółkiem odpowiednią liczbę. Każda odpowiedź jest dobra o ile jest prawdziwa.

1. Mam kłopoty z przystosowaniem się do ograniczeń narzuconych przez chorobę
zdecydowanie zdecydowanie
zgadzam się 1 2 3 4 5 nie zgadzam się

2. Z powodu swojego stanu zdrowie nie jestem w stanie robić tego, co najbardziej lubię.
zdecydowanie zdecydowanie
zgadzam się 1 2 3 4 5 nie zgadzam się

3. Choroba sprawia, że czasem czuję się niepotrzebny.
zdecydowanie zdecydowanie
zgadzam się 1 2 3 4 5 nie zgadzam się

4. Problemy ze zdrowiem sprawiają, że jestem bardziej zależny od innych niż tego chcę.
zdecydowanie zdecydowanie
zgadzam się 1 2 3 4 5 nie zgadzam się

5. Choroba sprawia, że jestem ciężarem dla swojej rodziny i przyjaciół.
zdecydowanie zdecydowanie
zgadzam się 1 2 3 4 5 nie zgadzam się

6. Mój stan zdrowia sprawia, że nie czuję się pełnowartościowym człowiekiem.
zdecydowanie zecydowanie
zgadzam się 1 2 3 4 5 nie zgadzam się

7. Nigdy nie będę samowystarczalnym w takim stopniu, w jakim chciałbym być.
zdecydowanie zdecydowanie
zgadzam się 1 2 3 4 5 nie zgadzam się

8. Myślę, że ludzie przebywający ze mną są często zakłopotani z powodu mojej choroby.
zdecydowanie zdecydowanie
zgadzam się 1 2 3 4 5 nie zgadzam się
